# Supplementary material for: Granulocytic Myeloid-Derived Suppressor Cells in Breast Milk (BM-MDSC) Correlate with Gestational Age and Postnatal Age and Are Influenced by Infant’s Sex
Source: Nutrients. 2020 Aug 25;12(9):2571. doi: 10.3390/nu12092571 (PMC7551580; doi:10.3390/nu12092571)
Supplement: Supplementary file 1 [file nutrients-12-02571-s001.pdf]

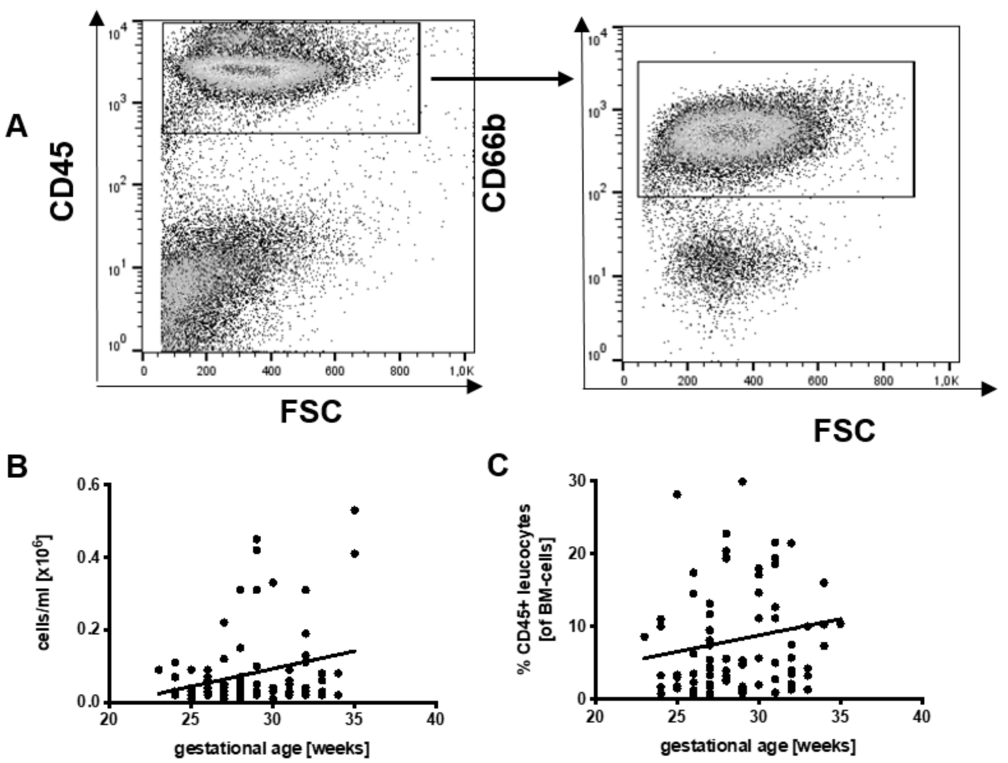

Figure S1. Quantification of cells in breast milk of preterm infants (dependent on gestational age). Milk cells were isolated from breast milk of mothers of preterm infants during the first five weeks after birth. Cells were counted on a hemocytometer and percentages of total CD45$^+$ leucocytes and GR-MDSC (of CD45$^+$ leucocytes) determined by flow cytometry. (**A**) Representative density plots show forward scatter (FSC) versus CD66b with gating strategy for BM-MDSC (**B** + **C**) the mean of total cell counts and percentages of total leucocytes over the first five weeks were calculated. Scatter diagrams showing total cell counts in milk (**B**) and percentages of leucocytes in milk (**C**), both in relation to gestational age. Regression lines show correlation between total cell counts (**B**) or percentages of leucocytes (**C**) and gestational age. N = 86, not significant, Spearman correlation.

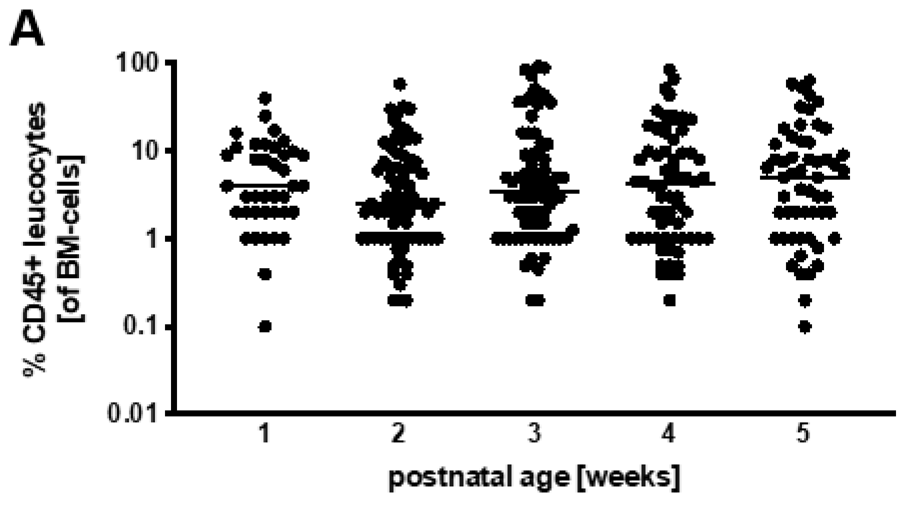

Figure S2. Quantification of $CD45^+$ leukocytes in breast milk of preterm infants (dependency on postnatal age). Milk cells were isolated from breast milk of mothers of preterm infants during the first five weeks of life. Percentages of $CD45^+$ leucocytes were determined by flow cytometry. (**A**) Scatter diagram showing the percentage of leucocytes from all cells in breast milk (BM) of mothers of preterm infants in postnatal weeks 1–5. N = 35–74, not significant; Kruskal-Wallis test and Dunn´s multiple comparison test.
